# Elevated Serum FGG Levels Prognosticate and Promote the Disease Progression in Prostate Cancer

**DOI:** 10.3389/fgene.2021.651647

**Published:** 2021-04-29

**Authors:** H. H. Peng, J. N. Wang, L. F. Xiao, M. Yan, S. P. Chen, L. Wang, K. Yang

**Affiliations:** ^1^Department of Urology, Chengdu Fifth People’s Hospital, Chengdu, China; ^2^Department of Urology, Tianjin Institute of Urology, The Second Hospital of Tianjin Medical University, Tianjin, China; ^3^Department of Reproductive Medicine, Xiangyang No. 1 People’s Hospital, Hubei University of Medicine, Xiangyang, China

**Keywords:** iTRAQ, ELISA, biomarkers, castration-resistant prostate cancer, FGG, cell proliferation

## Abstract

Castration-resistant prostate cancer (CRPC) threatens the health of men in general and no effective therapeutics currently exists for the treatment of CRPC. It is therefore of great importance to find a novel molecule that can be a biomarker and a therapeutic target for CRPC. First, we found that the serum fibrinogen gamma (FGG) levels in patients with CRPC were significantly higher than those with localized prostate cancer (PCa) through iTRAQ proteomics and ELISA experiments. Immunohistochemistry, quantitative real-time polymerase chain reaction and western blot also showed an increase of FGG expression in CRPC tissues and cells. Then we proved the proliferation, invasion and migration ability of CRPC cells were significantly reduced after FGG knockdown. The number of apoptotic cells increased at least sixfold after FGG silencing, and was observed in conjunction with an upregulation of p53, caspase 3, clea-caspase 3, and Bax, and a downregulation of Bcl2 and survivin. FGG knockdown in DU145 cells resulted in smaller xenografts than control cells in a mouse model. and we established that FGG is modulated by IL-6 which was increased in CRPC patients via phosphorylation of STAT3. The data suggests that FGG may be a potential therapeutic target and prognostic marker for CRPC.

## Introduction

Prostate cancer (PCa) is one of the most common malignancies in men and is a leading cause of cancer deaths (Attard et al., 2016). The incidence of PCa in China has risen rapidly over the past few decades. Radical prostatectomy is the first-line and most effective treatment of localized PCa. Nevertheless, patients with advanced PCa or relapsed disease have to accept androgen-deprivation therapy (ADT), which is standard therapy for metastatic PCa. ADT is initially effective, but within 2–3 years, PCa cells can adapt to low levels of androgen and ultimately progress to castration-resistant prostate cancer (CRPC) (De Las Pozas et al., 2018; Yu et al., 2019). Therefore, new therapeutic targets are urgently needed.

As a coagulation factor, fibrinogen is mainly synthesized by hepatocytes. Extrahepatic synthesis can also occur in epithelial cells and tumor cells. It is comprised of three pairs of non-identical polypeptide chains: two α-chains (FGA), two β-chains (FGB), and two γ-chains (FGG) interconnected with disulfide bonds (Repetto and De Re, 2017). There is increasing evidence suggesting that plasma fibrinogen is not only involved in the traditional hemostatic process, but also plays an important role in promoting inflammation and cancer progression (Palumbo and Degen, 2010). The extensive biological activity of fibrinogen and its degradation products can play important roles in tumor development by promoting cell proliferation, enhancing angiogenesis, stimulating capillary permeability, and regulating platelet activation (Balkwill and Mantovani, 2001).

Several studies have found increased levels of serum fibrinogen in diverse types of cancers, such as gastric cancer, colon cancer, liver cancer, ovarian cancer, and renal cell carcinoma (Pichler et al., 2013; Pedrazzani et al., 2016; Luo et al., 2017; Repetto and De Re, 2017; Ji et al., 2018). Furthermore, higher pretreatment plasma fibrinogen levels are associated with shorter overall survival and disease-free survival (Perisanidis et al., 2015). In PCa, some reports indicate that patients with high serum fibrinogen levels are more likely to have higher prostate-specific antigen (PSA) levels, Gleason scores, risk stratifications, and incidences of metastasis (Wang et al., 2016). It has been suggested that pretreatment levels of serum fibrinogen can be used as an independent prognostic index for ADT treatment (Ziaran et al., 2013). However, the effects of fibrinogen on PCa have not been elucidated. In this experiment, we used *in vitro* and *in vivo* methods to investigate the specific effects of fibrinogen on PCa.

## Materials and Methods

### Patient Selection

Patient serum samples were collected from the Second Hospital of Tianjin Medical University between 2014 and 2017. Twelve localized PCa patients were included in the initial screening and were divided into four groups (three patients per group). In each group, serum samples were collected at both the primary PCa and the CRPC stage. In total, eight samples were labeled and analyzed using iTRAQ. The differentially expressed proteins identified in the iTRAQ analyses were further validated by ELISA using serum samples collected from an additional 44 CRPC and 44 localized PCa patients(Table 1), none of the patients were receiving ADT treatment at that time. Informed consent was obtained from all the participants included in this study. This study had been approved by the Ethics Committee of the Second Hospital of Tianjin Medical University.

**TABLE 1:** Relationship between fibrinogen γ chain expression and patient clinical information.

Characteristics	*n*	Fibrinogen γ chain	expression	*p*-Value
		Low	High	
Total	88	58	30	
Age (years)				0.2988
≥60	70	48	22	
<60	18	10	8	
Gleason score				0.4747
≥6	76	49	27	
<6	12	9	3	
Metastasis				0.0034
Yes	29	13	16	
No	59	45	14	
CRPC				<0.001
Yes	44	20	24	
No	44	38	6	

### Proteomic Data Analysis

The Uniprot_homo database was used for protein identification. Mascot 2.3.02 was used for database searching. A protein must contain at least two unique spectra in order to qualify for quantitation. The quantitative protein ratios were weighted and normalized using the median ratio in Mascot. The significance level was set as *p*-values < 0.05 and fold-changes > 1.2.

#### ELISA Verification

Clinical blood specimen were tested for FGG with the ELISA kit (JL153, J&l Biological, China): patient whole blood samples were collected, incubated at room temperature for 2 h, and centrifuged at 1000× for 20 min to isolate the supernatant for use. A 1000 ng standard was used to set up a standard curve with the following concentrations: 1000, 500, 250, 125, 62.5, 31.25, 15.625, and 0 ng/mL. Blanks, standards, and sample wells were topped up to a final volume of 100 μL using PBS with 2% BSA. The membrane was incubated for 90 min at 37°C. The liquid was then discarded, and 100 μL of biotin-labeled antibody was added to each well. The membrane was incubated a second time for 1 h at 37°C. After washing three times, 100 μL of avidin-labeled horseradish peroxidase (HRP) was added to each well and the membrane was incubated at 37°C for 30 min. The membrane was then washed five times, and 90 μL of substrated solution (TMB) was added to each well and allowed to incubate at 37°C for 15 min in the dark. 50 μL of stop solution was then added to each well to terminate the reaction, and the optical density at 450 nm were immediated measured using a microplate reader (SPECTRA max plus 384). Contents below 21,943.03 ng/mL were considered as low expression and contents beyond 21,943.03 ng/mL were considered as high expression.

Cell culture medium: LNcap, C4-2, DU145, and PC3 cells were seeded in six-well plates at 2 × 10^4^ cells/well, the cell culture medium were collected after 72 h separately, centrifuged at 1000×*g* for 20 min and took the supernatant as test sample. Set standard wells, blank wells, and sample wells, and added 50 μL of each standard to the standard wells. (Concentrations of standards are: 8000, 4000, 2000, 1000, 500, 250 ng/mL.) Added 50 μL of the test sample to the sample wells and 50 μL of the sample dilution to the blank wells. A total of 100 μL of HRP-labeled detection antibody were added to all wells. Sealed the wells with a sealing plate and incubated at 37°C for 60 min. Washed the wells with washing solution (350 μL) for 5 × 1 min. Added 50 μL of substrate A and B to each well and incubated at 37°C for 15 min in the dark. Added 50 μL of stop solution to each well, and measured the OD value at 450 nm wavelength within 15 min.

### Cell Culture

LNcap, PC3, DU145, and C4-2 cells were provided by the Tianjin Institute of Urology. All the PCa cells were maintained in RPMI-1640 medium (Thermo Fisher Scientific, United States) supplemented with 10% fetal bovine serum (Life Technologies, Australia), 100 μg/mL penicillin, and 100 μg/mL streptomycin (Solarbio, China) in a humidified incubator with 5% CO_2_ at 37°C.

### Quantitative Real-Time Polymerase Chain Reaction

Total RNA was extracted using TRIzol^TM^ reagent (Life Technologies, United States) according to the manufacturer’s protocol. Complementary DNA (cDNA) was synthesized from total RNA using the HiFiScript cDNA Synthesis Kit (Cwbiotech, China). Quantitative real-time polymerase chain reaction (qRT-PCR) was performed using the Eco^TM^ Real-Time PCR System (Illumina, United States) and the parameters are as follows: 95°C for 10 min, 42 cycles of 95°C for 15 s, 60°C for 30 s, and 72°C for 15 s, and one final cycle of 95°C for 15 s, 60°C for 15 s, and 95°C for 15 s. The primers used for qRT-PCR are listed in Table 2. The 2^–ΔΔCt^ method was used to calculate relative gene expression levels.

**TABLE 2:** List of primers used for qRT-PCR.

Gene	Sequence (5′-3′)
**Gene-specific primer pairs for quantitative RT-PCR**	
**FGG**	
Forward	TACCAAGGTGGCACTTACTCA
Reverse	TCTGGTCTGACCTGTTTGGC
**STAT3**	
Forward	CATCCTGAAGCTGACCCAGG
Reverse	TATTGCTGCAGGTCGTTGGT
**Bcl2**	
Forward	ACTGGCTCTGTCTGAGTAAG
Reverse	CCTGATGCTCTGGGTAAC
**Caspase 3**	
Forward	TTAATAAAGGTATCCATGGAGAACA
Reverse	TAGAGTTCTTTTGTGAG
**Bax**	
Forward	GGGTTGTCGCCCTTTTCTAC
Reverse	GGTGAGGAGGCTTGAGGAGT
**Survivin**	
Forward	TGAACTTCAGGTGGATGAGGAGA
Reverse	GTCTAATCACACAGCAGTGGCAA
**P53**	
Forward	GCCCCTCCTCAGCATCTTAT
Reverse	AAAGCTGTTCCGTCCCAGTA
**GAPDH**	
Forward	AACAGCGACACCCACTCCTC
Reverse	GGAGGGGAGATTCAGTGTGGT

### Western Blot

Protein was extracted using the Total Protein Extraction Kit (Boster, Wuhan, China) and phenylmethylsulfonyl fluoride (100:1) (Solarboi, Beijing, China). The BCA assay was used to measure protein concentrations in order to normalize sample loading for separation on 10% SDS-PAGE. Separated proteins were transferred onto PVDF membranes and blocked with 5% BSA for 1 h. It was then incubated overnight at 4°C overnight with primary antibodies, washed with TBST for 3 × 15 min, and incubated for 1 h at 20°C with secondary antibodies. Protein bands were detected using enhanced chemiluminescence (ECL). GAPDH was used as the loading control. The antibodies used in this study are listed in Table 3.

**TABLE 3:** List of antibodies used in this study.

Antibody	Company	Host animal	Dilution
Anti-FGG	Abcom	Rabbit	1:800
Anti-STAT3	Proteintech	Mouse	1:800
Anti-pSTAT3	Proteintech	Mouse	1:800
Anti-P53	Proteintech	Mouse	1:1000
Anti-Bcl2	Proteintech	Mouse	1:500
Anti-survivin	Proteintech	Rabbit	1:500
Anti-caspase 3	Proteintech	Mouse	1:1000
Anti-Bax	Proteintech	Mouse	1:500
Anti-GAPDH	Proteintech	Rabbit	1:1500

### siRNA Transfection

Cells were seeded in six-well plates and were incubated with antibiotic-free medium half an hour before transfection. si-FGG, si-STAT3, and non-targeting control siRNA (si-control) (Synbio Technologies, China) were transfected in Opti-MEM medium (Invitrogen) using X-tremeGENE^TM^ siRNA Transfection Reagent (Roche, Germany) for 6 h according to the manufacturer’s instructions. The transfection medium was then replace with normal medium and the cells were cultured for an additional 48 h for subsequent experiments.

The sequence of si-FGG: 5′-GGUAGUUAUUGUCCA ACUA-3′, 5′-UAGUUGGACAAUAACUACC-3′.
The sequence of si-STAT3: 5′-GCACAAUCUACGAAGA AUCAA-3′. 5′-UUGAUUCUUCGUAGAUUGUGC-3′.

### Immunohistochemical Staining and Scoring

Paraffin-embedded sections for immunochemistry (IHC) were obtained from the Department of Pathology at the Second Hospital of Tianjin Medical University. After deparaffinization with Xylene, the slides were incubated with primary and secondary antibodies. The sections were stained with peroxidase (DAB). The staining intensity was evaluated on a three-point scale as follows: 0 (negative), 1 (weak), 2 (moderate), and 3 (strong). The percentage of stained cells were further divided and scored on a five-point scale: 0 (0%), 1 (1–25%), 2 (26–50%), 3 (51–75%), and 4 (76–100%). The final immunoreactivity score was calculated using the formula: IHC score = intensity score × percentage score. The primary antibody used for IHC was against FGG (dilution 1:50, 1001625, Proteintech, China).

### CCK-8 Assays

Cell proliferation was measured using the CCK-8 assay. A total of 3000 cells per well were seeded in a 96-well plate according to the manufacturer’s instructions. After 24 h, the cells were transfected with si-control or si-FGG. A total of 24, 48, and 72 h after transfection, CCK-8 reagents were added to the cells and incubated for 4 h in the humidified incubator. A microplate reader was used to measure the absorbance at a wavelength of 450 nm.

### Colony Formation Assays

The colony formation assay is another test for measuring cell proliferation. A total of 1000 cells per well were seeded in a six-well plate (Thermo Fisher Scientific, United States), then incubated at 37°C in 5% CO_2_ for 10 days, and the fresh medium was replaced according to the pH change of the medium until colonies shaped up (≥50 cells). Colonies were fixed with methanol for 15 min, dyed with crystal violet for 15 min, and then dried in the air. Finally, count the numbers of colonies.

### Cell Migration Assays

Transwell assays were performed using 24-well inserts (Corning, United States). A total of 72 h after transfection, 2 × 10^4^ cells in 200 μL of serum-free medium with 0.1% BSA were seeded in the upper chambers of the 24-well insert. A total of 600 μL of medium with 10% fetal bovine serum were added to the bottom chamber and co-cultured for another 48 h. The cells that have migrated to the bottom of the insert membrane were fixed with 4% formaldehyde, washed, and placed in 0.5% triton-100. The cells were counted under a microscope at 200× magnification.

### Wound-Healing Assays

The transfected cells were seeded into six-well plates at a density of 5 × 10^5^ cells per well. 48 h later, the cell layers were scratched with a pipette tip, washed, and incubated with a serum-free medium. Cell movements were observed and photographed at 24, 48, and 72 h after the scratch.

### Flow Cytometry Analysis

The Annexin V-FITC Apoptosis Analysis kit (Tianjin Sungene Biotech, China) was used to assess cell apoptosis. Cells were collected using EDTA-free pancreatic enzyme, washed with cold PBS, and resuspended in 1× binding buffer. Annexin V-FITC and PI solutions were added to the cells and incubated in the dark for 20 min at room temperature.

### Lentiviral Transduction

We purchased lentiviruses (Synbio Technologies, China) to create cell lines with FGG stably knocked down. DU145 cells were seeded in a 24-well plate at 5 × 10^4^ per well. Lentivirus containing sh-FGG (pLKO.1-EGFP-puro-hs-FGG-sh) and control lentivirus were used for transduction when the cells reached 80% confluence. Polybrene (8 μg/mL) was used to increase transduction efficiency. The medium was replaced with virus-free medium 24 h later. Puromycin (5 μg/mL) was used to select for stable knockdown cells.

The shRNA sequences are: 5′-CAGGAAAUAUAUA AUUCAA-3′; antisense 5′-UUGAAUUAUAUAUUUCCUG-3′.

### Animal Experiment

Four-week-old male athymic nude mice were randomly divided into two groups of five mice each, and a total of 1.0 × 10^7^ DU145 cells in 100 μLof PBS:Matrigel (1:1) were subcutaneously injected to establish xenograft tumors. Tumor size was measured 4 weeks after tumor formation, and tumor volume was calculated using the following formula: (shortest diameter)^2^ × longest diameter/2. All animal experiments were performed according to the proper guidelines for the use of experimental animals at Tianjin Medical University and were approved by the Experimental Animal Use Committee of Tianjin Medical University.

### Co-culture With Recombinant Human IL-6 and STAT3

IL-6 and STAT3 were diluted to a concentration of 0.1 mg/mL with axenic PBS. DU145 cells were seeded into six-well plates and the medium was replaced with serum-free medium before the addition of recombinant proteins. Cells were co-cultured in 50 or 100 ng/mL of IL-6/STAT3 (Proteintech, United States) for 24 or 48 h. The mRNA and protein were collected for downstream analysis.

### Statistical Analysis

Statistical analysis was performed using GraphPad Prism 7 and SPSS version 22. Student’s *t*-test or one-way ANOVA was used and a *p*-value less than 0.05 was considered statistically significant. All graphs are shown as mean ± SD and contains at least three independent experiments. The area under the ROC curve (AUC) was calculated to assess the diagnostic accuracy and identify the optimal cutoffs, sensitivity and specificity for CRPC. Standard criteria were used to assess the AUC.

## Results

### Serum FGG Is Higher in CRPC Patients Than in Patients With Localized PCa

**FIGURE 1:**
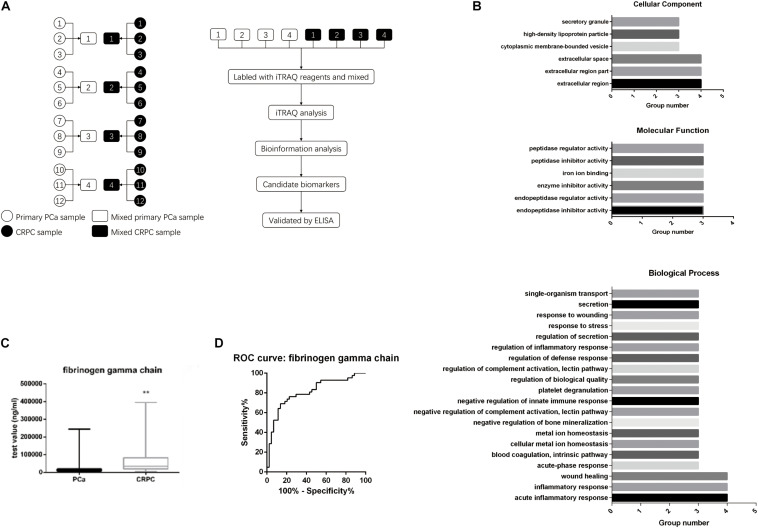
Serum FGG levels in CRPC patients are higher than patients with localized PCa. **(A)** Twelve patients were divided into four groups. Their serum was collected at both the primary PCa stage and when it has progressed to CRPC. Serum protein levels were analyzed with iTRAQ. **(B)** GO enrichment and KEGG pathway enrichment analyses for the differentially expressed genes. **(C)** Serum FGG levels in CRPC is higher than that of localized PCa. **(D)** Sensitivity and specificity of the ROC curve for using serum FGG as a diagnostic for CRPC. **p < 0.01.

We first selected 12 patients with localized PCa and divided them into four groups (three patients per group). We then collected their serum at both the primary PCa stage and when they relapsed as CRPC after ADT treatment (according to the AUA guidelines: rising PSA level and/or radiographic evidence of PCa progression despite medical or surgical castration). Eight serum samples were labeled and analyzed using iTRAQ (Figure 1A). We analyzed the proteins differentially expressed between localized PCa and CRPC. GO enrichment analysis and KEGG pathway enrichment analysis for the differentially expressed proteins were performed in each group. GO terms that were significant in at least three groups were identified (listed in Figure 1B). To further identify the important differentially expressed proteins, we narrowed down the candidates to only those with significant and consistent changes in at least three of the four groups. For proteins that can be identified by the same set of peptides, only those included in the UniProtKB/Swiss-Prot database were chosen. Proteins without commercially available ELISA kits were excluded from downstream validation. In the end, eight proteins were identified by these criteria for further validation: alpha-2-HS-glycoprotein, actin cytoplasmic 2, actin cytoplasmic 1, calreticulin, keratin, type I cytoskeletal 10, coagulation factor IX, fibrinogen gamma chain, and hemoglobin subunit beta. In order to verify the candidates identified above, we examined the expression levels of these proteins in the serum samples of 44 CRPC and 44 localized PCa patients using ELISA kits. A summary of the patient characteristics are listed in Table 1. We found that the expression of fibrinogen gamma chain (FGG) was consistently significantly increased in the serums of CPRC patients when compared with localized PCa patients (*p* < 0.01) (Figure 1C). In addition, we used serum FGG levels to establish an ELISA-based assay for the detection of CRPC. Samples can be discriminated using a serum FGG detection value between ∼ 19,748.73 and 24,137.33 ng/mL, preferably with 21,943.03 ng/mL as the threshold. If the serum FGG level is greater than or equal to the threshold, the patient has progressed to CRPC; if the serum FGG level is less than the threshold, the patient has not progressed to CRPC. In the present 88 samples, the sensitivity of the ROC curve for diagnosing CRPC using our assay is 69% and the specificity is 86.4% (Figure 1D).

### Upregulation of FGG in CRPC Tissues and Cells

**FIGURE 2:**
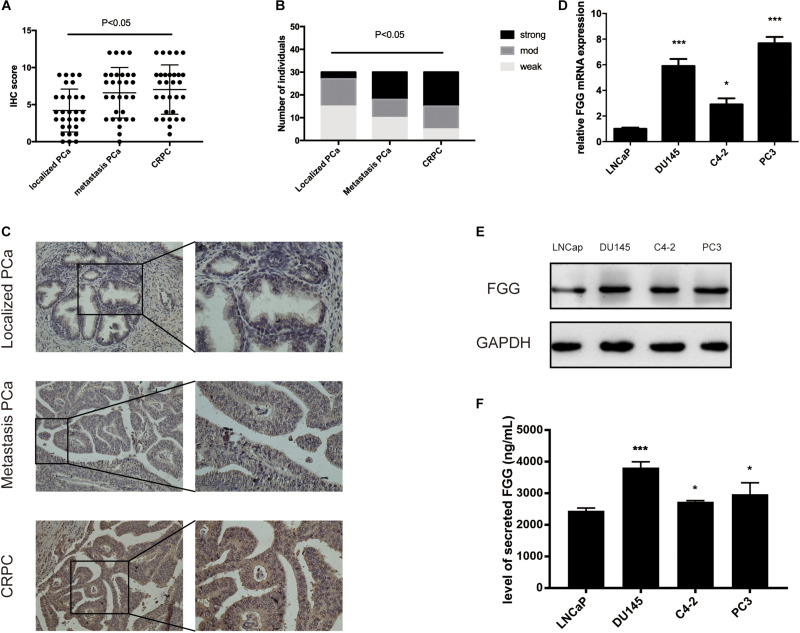
Upregulation of FGG expression in CRPC tissues and cells. **(A)** FGG IHC scores of localized PCa, metastasis PCa, and CRPC tissues. **(B)** FGG staining intensity in localized PCa, metastasis PCa, and CRPC tissues. **(C)** Representative IHC staining images of various tissues. **(D)** Relative FGG mRNA expression in PCa cell lines. **(E)** Relative FGG protein expression in PCa cell lines. **(F)** Level of secreted FGG in PCa cell lines. *p < 0.05, ***p < 0.001.

By analyzing patient serum and clinical data, we found that FGG is not only significantly elevated in CRPC patients, but also in patients with metastatic PCa. We wondered if PCa tissues and cells show the same tendency. Paraffin-embedded localized PCa tissues (*n* = 30), metastasis PCa tissues (*n* = 30), and CRPC tissues (*n* = 30) were selected for FGG IHC staining. Results indicated that localized PCa tissues had significantly lower IHC scores and weaker staining intensities than metastasis PCa and CRPC tissues (Mann–Whitney test; *p* < 0.01) (Figures 2A,B). Representative staining images are shown in Figure 2C. Furthermore, the mRNA and protein levels of FGG in LNcap, C4-2, DU145, and PC3 cells were detected by RT-qPCR and western blot. FGG expression in LNcap cells was the lowest compared to the other PCa cell lines (C4-2, DU145, and PC3). Cells lines that lack androgen receptor (PC3 and DU145) had the highest expression levels of FGG (Figures 2D,E). In addition, we tested the secretion level of FGG in each cell line. As shown in Figure 2F, PC3 cells and DU145 cells culture medium had the most secretion of FGG. As such, PC3 and DU145 cells were chosen for further studies.

### FGG Knockdown Reduces the Proliferation, Migration, and Invasion Capabilities of PCa Cells

**FIGURE 3:**
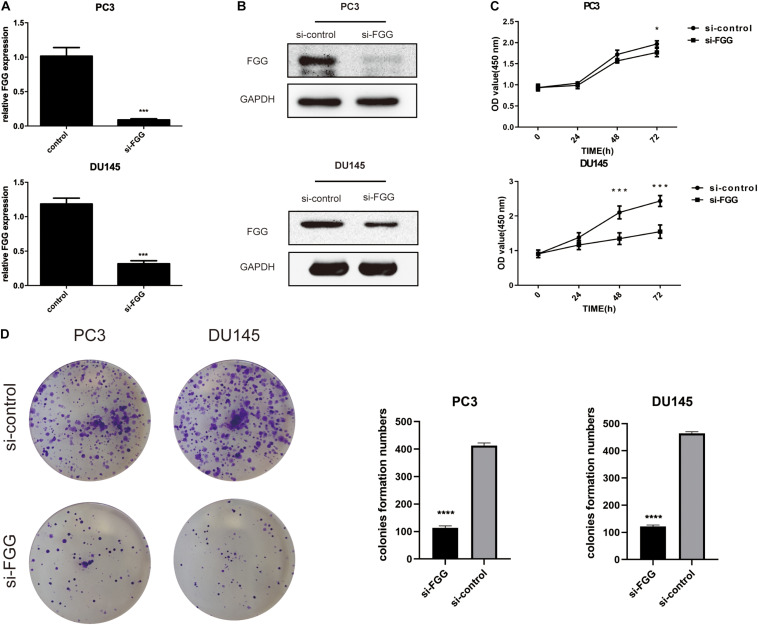
FGG knockdown reduces the proliferation capabilities of PCa cells. **(A)** qRT-PCR and **(B)** western blot analyses indicate that si-FGG significantly suppressed FGG mRNA and protein expression. **(C)** CCK8 experiments demonstrate that FGG knockdown decreased cell proliferation in PC3 and DU145 cells compared to control cells. **(D)** Colony formation assays shows that FGG knockdown significantly decreased cell proliferation in PC3 and DU145 cells. *p < 0.05, ***p < 0.001, ****p < 0.0001.

**FIGURE 4:**
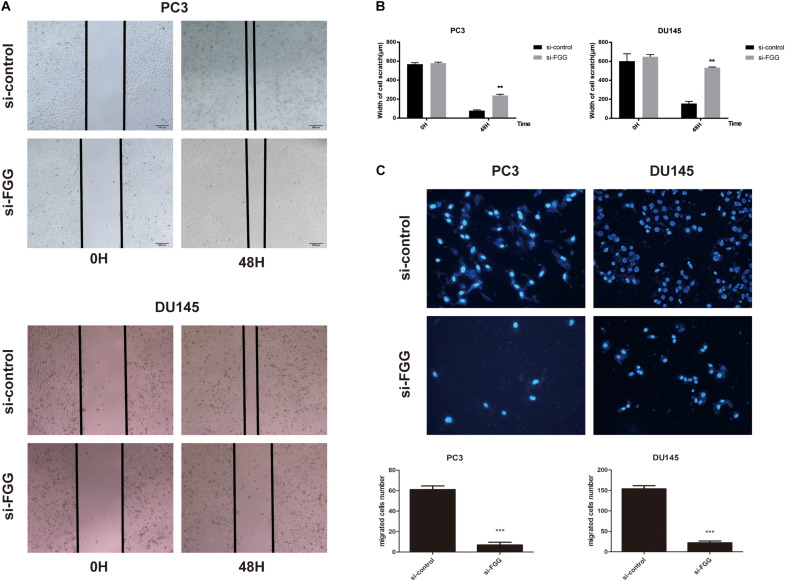
FGG knockdown reduces the migration and invasion capabilities of PCa cells. **(A)** Wound-healing assays demonstrate that FGG knockdown decreases cell migration in PC3 and DU145 cells compared to control cells. **(B)** Quantification of the cell scratch. **(C)** DAPI staining and cell counting from transwell assays indicate that FGG knockdown reduces the invasiveness of PC3 and DU145 cells. **p < 0.01, ***p < 0.001.

Since FGG is highly expressed in PC3 and DU145 cells, we used small interfering RNA (siRNA) to knock down the gene expression in these two cell lines. si-FGG or si-control was transfected into both cell lines. Transfection efficiency was confirmed by qRT-PCR and western blot as the expression of FGG decreased by more than 70% (Figures 3A,B). The effect of FGG on cell proliferation was verified using the CCK8 assay and Colony formation assay. FGG knockdown significantly reduced the cell proliferation of DU145 and PC3 cells (Figure 3C,D). We then explored the effects of FGG on the invasion and migration capabilities of PCa cells. In wound-healing assays, knockdown of FGG significantly decreased cell mobility (Figures 4A,B). Transwell assays showed similar results. Fewer si-FGG cells passed through the membrane than cells transfected with si-control (Figure 4C). These results are consistent with previous research showing fibrinogen as a key determinant of tumor cell metastatic potential (Palumbo et al., 2000).

### Downregulation of FGG Increases Cell Apoptosis

**FIGURE 5:**
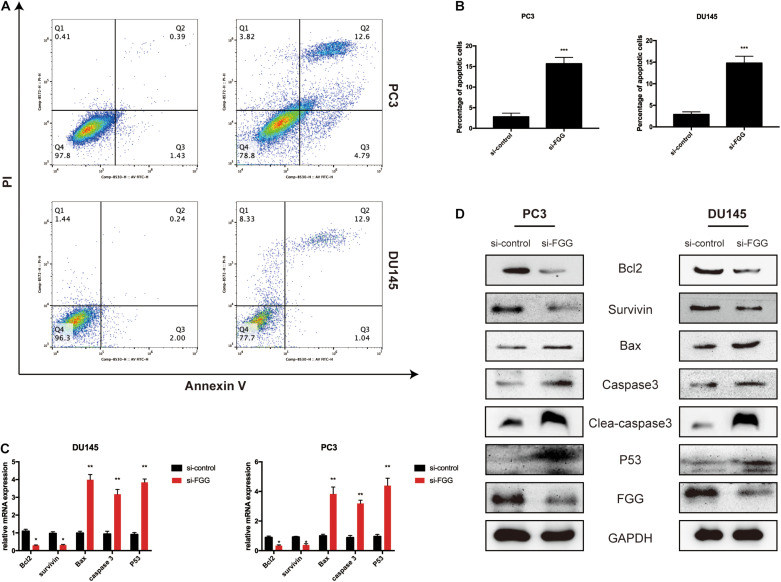
Downregulation of FGG increases cell apoptosis. **(A)** Flow cytometry analyses revealed that the apoptosis ratio increased significantly after FGG knockdown in PC3 and DU145 cells. **(B)** The quantification of apoptotic ratio. **(C)** The relative mRNA expressions of apoptosis-associated factors in si-control/si-FGG PC3 and DU145 cells. **(D)** The relative protein expressions of apoptosis-associated factors in si-control/si-FGG PC3 and DU145 cells. *p < 0.05, **p < 0.01, ***p < 0.001.

Next, we assessed whether proliferation inhibition in si-FGG cells is associated with cell apoptosis. A flow cytometry experiment with Annexin V and PI staining demonstrated that the apoptosis ratio had increased by at least sixfold in si-FGG cells compared to si-control PC3 and DU145 cells (Figures 5A,B). This suggests that FGG influences cell apoptosis to a certain extent. To further validate our findings, we assessed the changes to additional apoptosis-associated factors following si-FGG treatment. The results from qPCR and western blots show that the mRNA and protein levels of survivin and Bcl2 were downregulated, while the mRNA and protein levels of caspase 3, clea-caspase 3, Bax, and P53 were upregulated in si-FGG PC3 cells. The same trend can also be observed in si-FGG DU145 cells (Figures 5C,D).

### FGG Knockdown Inhibits PCa Cell Proliferation *in vivo*

**FIGURE 6:**
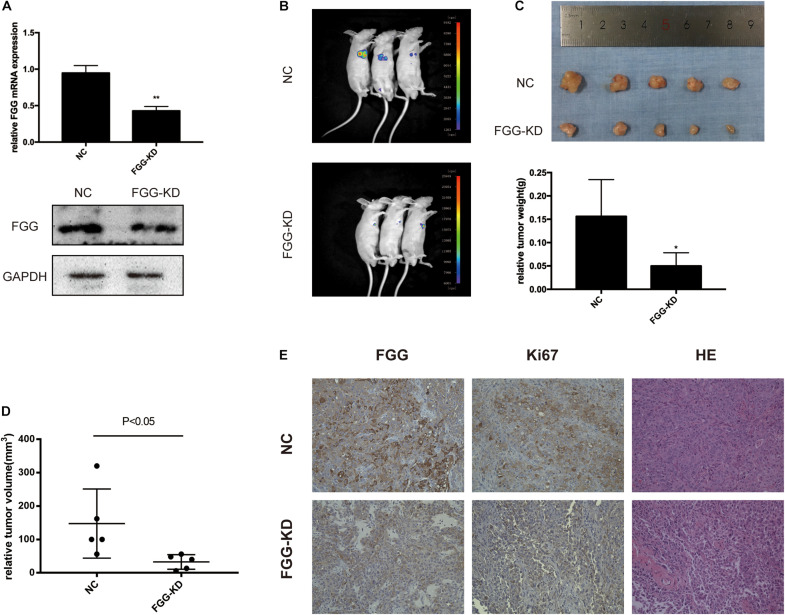
FGG knockdown inhibits PCa cell proliferation *in vivo*. **(A)** The efficiency of the shRNA. **(B)** Bioluminescence images of the resultant DU145 tumors in the NC and FGG-KD groups. **(C)** Subcutaneous tumors were excised and weighed 4 weeks after inoculation. **(D)** Relative tumor volumes of the NC and FGG-KD groups. **(E)** Representative IHC and HE staining images of the NC and FGG-KD subcutaneous tumors. *p < 0.05, **p < 0.01.

To further evaluate the function of FGG on tumor growth *in vivo*, we established a xenograft mouse model using DU145 cells that stably express shRNA knocking down FGG expression (KD) or an empty vector control (NC). qRT-PCR and western blot experiments verified that the shRNA efficiency was greater than 60% (Figure 6A). A total of 1 × 10^7^ cells were subcutaneously implanted under the arm of nude mice. Bioluminescence images showed a substantially lower luciferase signal in the FGG-KD group than in the NC group 4 weeks after inoculation (Figure 6B). The size of the FGG-KD xenograft tumors was significantly smaller than those in the NC group (Figure 6C), and the tumor weights in the FGG-KD group was also remarkably lower than the NC group (Figure 6D). Immunohistochemical staining of the dissected tumor tissues showed that the FGG staining was weakened and the number of Ki67 positive cells were significantly decreased in the FGG-KD group (Figure 6E).

### Elevated Fibrinogen After ADT Is Mediated by the IL-6/STAT3 Pathway

**FIGURE 7:**
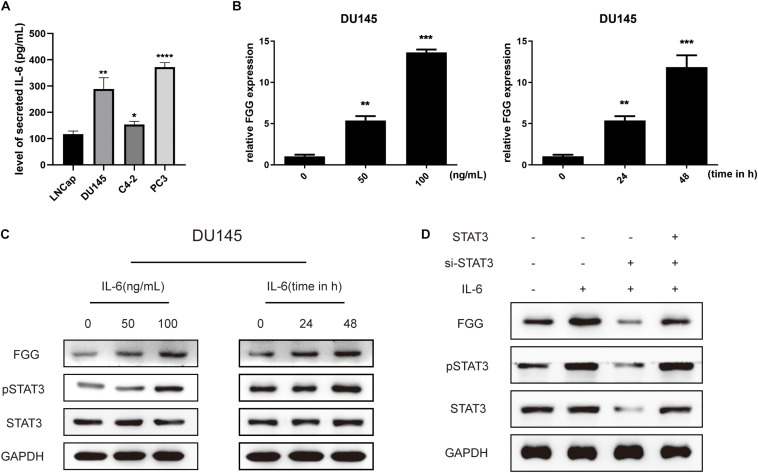
Elevated fibrinogen following ADT is mediated by the IL-6/STAT3 pathway. **(A)** Level of secreted IL-6 in PCa cell lines. **(B)** Relative FGG mRNA expression levels 48 h after treatment with 0, 50, or 100 ng/mL of IL-6, and also at 0, 24, and 48 h after treatment with 100 ng/mL of IL-6. **(C)** Relative protein levels of FGG, pSTAT3, and STAT3 48 h after treatment with 0, 50 or 100 ng/mL of IL-6, and also at 0, 24, and 48 h after treatment with 100 ng/mL of IL-6. **(D)** Relative protein expression levels of FGG, pSTAT3, and STAT3 after addition of IL-6, si-STAT3, or exogenous STAT3. *p < 0.05, **p < 0.01, ***p < 0.001, ****p < 0.0001.

Studies have shown that the IL-6/STAT3 signaling pathway is associated with PCa progression, and that serum levels of IL-6 are higher in patients with metastatic PCa and CRPC compared to those with localized PCa. Further studies have found that anti-androgen therapy or knockdown of the AR gene results in the up-regulation of IL-6 expression (Lou et al., 2000; Paule et al., 2007; Schroeder et al., 2013). We thus explored whether elevated FGG is associated with the IL-6/STAT3 signaling pathway during ADT. First, we tested the expression of IL-6 in different PCa cells, and found that the synthesis of IL-6 in hormone-resistant cells (DU145, PC3, and C4-2) was significantly higher than that of hormone-sensitive cells (LNCap) (Figure 7A). Then, Recombinant human IL-6 protein was added to the culture medium of DU145 cells. A total of 48 h after incubation, the relative mRNA expression levels of FGG increased with increasing IL-6 concentrations (0, 50, and 100 ng/mL). The amount of FGG mRNA expression also increased over time (0, 24, and 48 h) when the same concentration of IL-6 (100 ng/mL) was used (Figure 7B). Western blots also showed that the levels of FGG and phosphorylated STAT3 (pSTAT3) increased with exposure to IL-6, while total levels of STAT3 remained relatively steady (Figure 7C). Knockdown of STAT3 in DU145 cells supplemented with IL-6 resulted in a significant decrease in FGG expression. When recombinant human STAT3 was exogenously added, the reduction in FGG expression is reversed (Figure 7D). Taken together, the results indicate that FGG is a downstream target gene regulated by the IL-6/STAT3 pathway. We hypothesize that the inhibition of AR signaling promotes IL-6 expression in PCa during ADT, and elevated IL-6 increases FGG expression by regulating STAT3 signaling.

## Discussion

Hyperfibrinogenemia is observed across different cancer types and has attracted attention of many researchers. Fibrinogen levels are significantly correlated with tumor size, depth of invasion, lymph node metastasis, and TNM stages in gastric cancer (Yu et al., 2016). In colon cancer, Steinbrecher et al. (2010) analyzed fibrinogen mutant mice lacking the leukocyte integrin receptor αmβ2 binding motif and found that αMβ2-mediated engagement of fibrinogen was involved in local inflammation and adenoma formation. In Lewis lung cancer and B16-BL6 melanoma mice, the diffusion and metastasis of tumor cells are strongly inhibited in the absence of fibrinogen. This effect can be further enhanced by hirudin (a specific thrombin inhibitor) (Palumbo et al., 2000). Similarly in gallbladder tumor cells, fibrinogen can promote migration and metastasis by increasing the expression of vimentin, decreasing the expression of E-cadherin, and promoting the process of epithelial-mesenchymal transition (EMT) (Shu et al., 2014). These observations all suggest that fibrinogen plays an important role in tumor progression.

Previous studies have reported that the level of fibrinogen increases gradually during ADT in PCa. A high level of plasma fibrinogen concentration is also negatively correlated with cancer-specific and overall survival (Thurner et al., 2015). Fibrinogen is composed of three different polypeptide chains, of which the gamma chain (FGG) is the main peptide. By collecting clinical blood samples from PCa patients at different disease stages, we detected eight proteins that were differentially expressed in patients with CRPC compared to those with localized PCa. Further analysis revealed that FGG expression was significantly elevated in patients with CRPC. This elevated FGG can be detected by ELISA and can be used to help diagnose of CRPC. This elevated FGG expression is also verified in PCa tissues and cells.

Sahni et al. (2008) reported that fibrinogen promotes the proliferation of PCa cells by enhancing the effects of fibroblast growth factor-2 (FGF-2). Our experiments verified the proliferative effects of FGG. In addition, we also found that FGG promotes the migration and invasion abilities of PCa cells. Flow cytometry further revealed that FGG knockdown resulted in at least a sixfold increase in apoptotic cells. These FGG knocked-down cells had a lower expression of survivin and Bcl2 and a higher expression of Bax, P53, and caspase 3. Furthermore, we verified the effect of FGG on tumor growth *in vivo*. We found that subcutaneous xenografts of FGG knocked-down cells were significantly smaller than tumors from control cells. Moreover, We conducted IHC and HE staining of the dissected tumor tissues. IHC results showed that the FGG staining was weakened and the number of Ki67 positive cells were significantly decreased in the FGG-KD group, all of those indicating that tumor growth has been impactful inhibited.

Finally, there was evidence in the literature that serum IL-6 levels would increase during ADT. It has been suggested that high levels of IL-6 (>7 pg/mL) can promote malignant progression from hormone-sensitive to hormone-refractory PCa (Nakashima et al., 2000). The binding of IL-6 to its receptor can activate several intracellular signaling cascades, including the JAK-STAT and MAPK pathways (Ben Jemaa et al., 2012). Liu et al. (2014) reported that the IL-6/STAT3 axis is involved in enzalutamide resistance in PCa. And studies have found that in mice bearing TC1 murine prostate tumors, whether they were treated with antiandrogen therapy or silencing of AR, the expression of IL-6 and STAT3 in mouse tumors would increase. In addition, silencing the expression of AR in PCa cells by RNA interference, it was found that AR knockdown resulted in more powerful STAT3 activation, thereby compensating for the silenced AR and promoting tumor growth. As we found that the synthesis of IL-6 in hormone-resistant cells was significantly higher than that of hormone-sensitive cells, We explored whether elevated FGG during ADT is associated with increased IL-6 expression. By adding recombinant human IL-6 to the culture medium, we found that IL-6 can significantly increase the expression of FGG by mediating the phosphorylation of STAT3. Then we used siRNA to knockdown STAT3, and found by western blotting that the FGG is downregulated despite IL-6 stimulation. The addition of recombinant human STAT3 exogenously can reverse the decrease of FGG expression. We speculate that during the treatment of ADT, the function of AR was inhibited, IL-6 and STAT3 were activated for compensation, and the expression of IL-6 and STAT3 promoted the increased synthesis of FGG, thereby promoting the growth of the prostate tumor.

Taken together, our study demonstrates the specific changes in serum FGG levels in CRPC patients and systematically elucidated the effects of FGG on tumor progression *in vitro* and *in vivo*. For the first time, we developed an ELISA assay for the detection of CRPC based on serum FGG levels. This suggests that FGG may act as an auxiliary diagnostic indicator and a new therapeutic target for CRPC.

## Data Availability Statement

The raw data supporting the conclusions of this article will be made available by the authors, without undue reservation.

## Ethics Statement

The studies involving human participants were reviewed and approved by the Medical Ethics Committee of the Second Hospital of Tianjin Medical University. The patients/participants provided their written informed consent to participate in this study. The animal study was reviewed and approved by the Medical Ethics Committee of the Second Hospital of Tianjin Medical University. Written informed consent was obtained from the individual(s) for the publication of any potentially identifiable images or data included in this article.

## Author Contributions

HP completed the conception and implementation of the whole article. JW addressed the mass spectrum data analysis and helped complete experimental ideas. LX helped complete the experimental design and part of the experiments. MY helped supplement the experiments and the article. SC and LW guided the completion of part of experiments and data collection and analysis. All authors contributed to the article and approved the submitted version.

## Conflict of Interest

The authors declare that the research was conducted in the absence of any commercial or financial relationships that could be construed as a potential conflict of interest.

## References

[B1] Attard G. Parker C. Eeles R. A. Schröder F. Tomlins S. A. Tannock I. (2016). Prostate cancer. *Lancet* 387 70–82. 26074382 10.1016/S0140-6736(14)61947-4

[B2] Balkwill F. Mantovani A. (2001). Inflammation and cancer: back to Virchow? *Lancet* 357 539–545. 10.1016/s0140-6736(00)04046-0 11229684

[B3] Ben Jemaa A. Sallami S. Ramarli D. Colombatti M. Oueslati R. (2012). The proinflammatory Cytokine, IL-6, and its interference with bfgf signaling and Psma in prostate cancer cells. *Inflammation* 36 643–650. 10.1007/s10753-012-9586-7 23250823

[B4] De Las Pozas A. Reiner T. De Cesare V. Trost M. Perez-Stable C. (2018). Inhibiting multiple deubiquitinases to reduce androgen receptor expression in prostate cancer cells. *Sci. Rep.* 8:13146. 10.1038/s41598-018-31567-3 PMC6120934 30177856

[B5] Ji R. Ren Q. Bai S. Wang Y. Zhou Y. (2018). Prognostic significance of pretreatment plasma fibrinogen in patients with hepatocellular and pancreatic carcinomas: a meta-analysis. *Medicine (Baltimore)* 97 e10824. 10.1097/md.0000000000010824 29923974 PMC6023750

[B6] Liu C. Zhu Y. Lou W. Cui Y. Evans C. P. Gao A. C. (2014). Inhibition of constitutively active Stat3 reverses enzalutamide resistance in LNCaP derivative prostate cancer cells. *Prostate* 74 201–209. 10.1002/pros.22741 24307657 PMC4437226

[B7] Lou W. Ni Z. Dyer K. Tweardy D. J. Gao A. C. (2000). Interleukin-6 induces prostate cancer cell growth accompanied by activation of Stat3 signaling pathway. *Prostate* 42 239–242. 10.1002/(sici)1097-0045(20000215)42:3<239::aid-pros10>3.0.co;2-g 10639195

[B8] Luo Y. Kim H. S. Kim M. Lee M. Song Y. S. (2017). Elevated plasma fibrinogen levels and prognosis of epithelial ovarian cancer: a cohort study and meta-analysis. *J. Gynecol. Oncol.* 28:e36. 10.3802/jgo.2017.28.e36 PMC5391395 28382799

[B9] Nakashima J. Tachibana M. Horiguchi Y. Oya M. Ohigashi T. Asakura H. (2000). Serum interleukin 6 as a prognostic factor in patients with prostate cancer. *Clin. Cancer Res.* 6 2702–2706. 10914713

[B10] Palumbo J. S. Kombrinck K. W. Drew A. F. Grimes T. S. (2000). Fibrinogen is an important determinant of the metastatic potential of circulating tumor cells. *Blood* 96 3302–3309. 10.1182/blood.v96.10.3302 11071621

[B11] Palumbo S. J. Degen L. J. (2010). Mechanisms coupling the hemostatic system to colitis-associated cancer. *Thromb. Res.* 125 S39–S43. 20434003 10.1016/S0049-3848(10)70011-6

[B12] Paule B. Terry S. Kheuang L. Soyeux P. Vacherot F. de la Taille A. (2007). The NF-kappaB/IL-6 pathway in metastatic androgen-independent prostate cancer: new therapeutic approaches? *World J. Urol.* 25 477–489. 10.1007/s00345-007-0175-6 17541600

[B13] Pedrazzani C. Mantovani G. Salvagno G. L. Baldiotti E. Ruzzenente A. Iacono C. (2016). Elevated fibrinogen plasmalevel is not an independent predictor of poor prognosis in a large cohort of Western patients undergoing surgery for colorectal cancer. *World J. Gastroenterol.* 22 9994–10001. 10.3748/wjg.v22.i45.9994 28018106 PMC5143766

[B14] Perisanidis C. Psyrri A. Cohen E. E. Engelmann J. Heinze G. Perisanidis B. (2015). Prognostic role of pretreatment plasma fibrinogen in patients with solid tumors: a systematic review and meta-analysis. *Cancer Treat. Rev.* 41 960–970. 10.1016/j.ctrv.2015.10.002 26604093

[B15] Pichler M. Hutterer G. C. Stojakovic T. Mannweiler S. Pummer K. Zigeuner R. (2013). High plasma fibrinogen level represents an independent negative prognostic factor regarding cancer-specific, metastasis-free, as well as overall survival in a European cohort of non-metastatic renal cell carcinoma patients. *Br. J. Cancer* 109 1123–1129. 10.1038/bjc.2013.443 23922109 PMC3778296

[B16] Repetto O. De Re V. (2017). Coagulation and fibrinolysis in gastric cancer. *Ann. N. Y. Acad. Sci.* 1404 27–48. 10.1111/nyas.13454 28833193

[B17] Sahni A. Simpson-Haidaris P. J. Sahni S. K. Vaday G. G. Francis C. W. (2008). Fibrinogen synthesized by cancer cells augments the proliferative effect of fibroblast growth factor-2 (FGF-2). *J. Thromb. Haemost.* 6 176–183. 10.1111/j.1538-7836.2007.02808.x 17949478

[B18] Schroeder A. Herrmann A. Cherryholmes G. Kowolik C. Buettner R. Pal S. (2013). Loss of androgen receptor expression promotes a stem-like cell phenotype in prostate cancer through Stat3 signaling. *Cancer Res.* 74 1227–1237. 10.1158/0008-5472.can-13-0594 24177177 PMC4539262

[B19] Shu Y. j. Weng H. Bao R. f. Wu X. s. Ding Q. (2014). Clinical and prognostic significance of preoperative plasma hyperfibrinogenemia in gallbladder cancer patients following surgical resection: a retrospective and in vitro study. *BMC Cancer* 14:566. 10.1186/1471-2407-14-566 25096189 PMC4131047

[B20] Steinbrecher K. A. Horowitz N. A. Blevins E. A. Barney K. A. Shaw M. A. Harmel-Laws E. (2010). Colitis-associated cancer is dependent on the interplay between the hemostatic and inflammatory systems and supported by integrin alpha(M)beta(2) engagement of fibrinogen. *Cancer Res.* 70 2634–2643. 10.1158/0008-5472.can-09-3465 20233870 PMC4288842

[B21] Thurner E. M. Krenn-Pilko S. Langsenlehner U. Stojakovic T. Pichler M. Gerger A. (2015). The association of an elevated plasma fibrinogen level with cancer-specific and overall survival in prostate cancer patients. *World J. Urol.* 33 1467–1473. 10.1007/s00345-014-1459-2 25475065

[B22] Wang Y. Yin W. Wang Z. Huang J. Pan J. Zhu Y. (2016). Pretreatment plasma fibrinogen as an independent prognostic indicator of prostate cancer patients treated with androgen deprivation therapy. *Prostate Cancer Prostatic Dis.* 19 209–215. 10.1038/pcan.2016.6 26951714

[B23] Yu W. Li J. Wang Q. Wang B. Zhang L. Liu Y. (2019). Targeting POH1 inhibits prostate cancer cell growth and enhances the suppressive efficacy of androgen deprivation and docetaxel. *Prostate* 79 1304–1315. 10.1002/pros.23838 31212367

[B24] Yu W. Wang Y. Shen B. (2016). An elevated preoperative plasma fibrinogen level is associated with poor overall survival in Chinese gastric cancer patients. *Cancer Epidemiol.* 42 39–45. 10.1016/j.canep.2016.03.004 27010728

[B25] Ziaran S. Goncalves F. M. Breza J. Sr. (2013). Patients with prostate cancer treated by ADT have significantly higher fibrinogenemia than healthy control. *World J. Urol.* 31 289–292. 10.1007/s00345-012-0926-x 22898989

